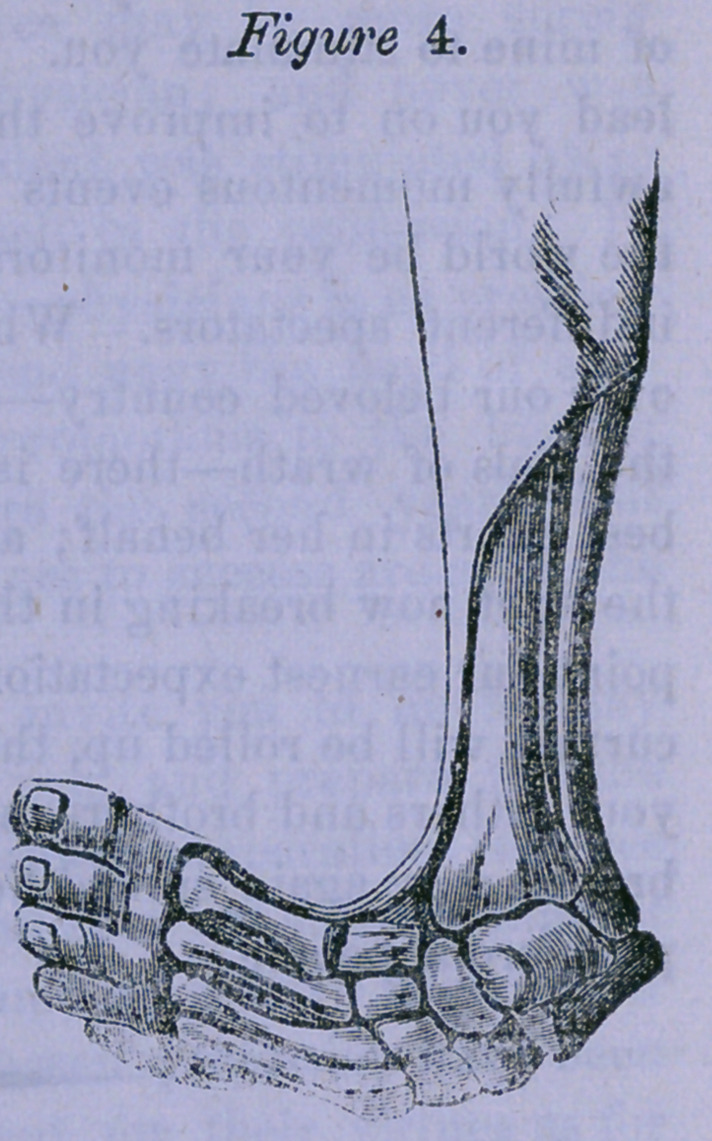# Report on Orthopedic Surgery, Made at the Annual Meeting of the Illinois State Medical Society, Convened in Chicago, May 3d, 1864

**Published:** 1864-11

**Authors:** David Prince

**Affiliations:** Jacksonville, Ill.


### REPORT ON ORTHOPEDIC SURGERY,

### MADE AT THE ANNUAL MEETING OF THE ILLINOIS STATE MEDICAL SOCIETY, CONVENED IN CHICAGO, MAY 3D, 1864.

By DAVID PRINCE, M. D., Jacksonville, Ill.

*(Continued from last month.)*

As the various species of congenital Talipes are similar to the corresponding deformities developed subsequently to birth from derangements of innervation, it is fair to infer that, in most cases, a similar derangement of innervation has existed during fœtal life. This conjecture is rendered more probable by dissection, which shows that the bones of the tarsus have their proper forms until they are afterward slightly changed in figure by the great pressure to which they are subjected in walking.

This change is much less than a superficial glance would lead one to suppose, there being nowhere a complete dislocation, but only a sliding a little further than the normal length of the ligaments permits.

*Figure 4.*

The following figure, (Fig. 4,) taken from "Little on the Nature and Treatment of the Deformities of the Human Frame," sufficiently illustrates this point.

The relative importance of paralysis and spasm in the production of this and other deformities will be differently appreciated by different minds standing in opposite positions. The quotations from Bauer,(*), representing the older pathology, and from Barwell, (†), representing the newer, illustrate this point:

Dr. Bauer thinks (p. 12) that contraction of the sural muscles (the muscles ending in the tendo achilles) generally the chief cause of the extension of the foot in Talipes equinus. He makes no account of the doubling up of the foot at the medio tarsel articulation so carefully explained by Little and Barwell, and equally with Barwell omits to mention the calcaneo-metatarsal and calcaneo-phalangial muscles as elements in the etiology.

Referring the disease to the shortened muscles, he says: "As a general thing, the contracted muscles have lost all susceptibility of being acted upon by the galvanic current,

(*). Lecture on Orthopedic Surgery, by Louis Bauer, M. D. Lindsay & Blackiston: Philadelphia, 1864.

(†). The Treatment of Club-Foot without the Division of Tendons, by Mr. Richard Barwell, &c. London: 1863.

yet their powerful extension gives rise to unbearable pain. This fact seems to demonstrate that the muscular structure is in a state of contraction to the extent of its capacity, or the substituted tissue is devoid of all contractile" (expansive) "power. It is certain that innervation has not been entirely lost while pain can be provoked by extension."

In the conditions referred to in this paragraph, the occurrence of pain may perhaps be better explained by bearing in mind that the muscles concerned have for the time acquired the conditions of ligaments.

We know well enough that the ligaments are susceptible of acute pain when over stretched. When a muscle, therefore, which has lost its function from loss, change or paralysis of its muscular substance, is pulled further than its investments of white fibrous tissue will permit, without injury to its habitual physical condition, it is in close analogy with an over stretched ligament, and it should be the seat of pain, the same as if it had originally been a ligament.

The following additional quotation is a further illustration of the spasmodic pathology:

"After the division of the tendons many months may elapse before the galvanic current makes any impression, and in some instances the contractility of the muscles is gone forever."

If the division of tendons is all that is done, the shortening ought to go on still more. It is probably the subsequent movements, effected in the course of the treatment, that restore the susceptibility to the galvanic current.

Dr. Bauer finds an advocate for the doctrine of tonic spasm as the cause of Talipes equinus in Dr. Joseph Pancoast,* of

* Dr. Joseph Pancoast, of Philadelphia, claims that the elevation of the heel in Talipes equinus is owing to the contraction of the soleus while the gastrocnemius remains flaccid, and he accordingly divides the soleus muscle by passing a knife in under the gastrocnemius, instead of the usual easy method of dividing the tendo Achillis.

It is found in any confirmed case of Talipes equinus or T. equino varus, that the soleus is rigid and incapable of extension, while the gastrocnemius is yielding. Dr Pancoast is therefore of opinion that the soleus is the author of the mischief.

The fact has another explanation. When a muscle contracts with such

Philadelphia, who thinks that of the three muscles uniting to make the tendo achillis, only the soleus is inordinately contracted, and accordingly he only divides the soleus in the treatment. This is done by passing the bistoury under the gastrocneimus and cutting the soleus just as it becomes tendinous and unites with the gastrocneimus; the edge of the knife being carried towards the bones for this purpose.

It would be wrong, however, to leave the reader with the impression that Dr. Bauer considers spasm the uniform cause of Talipes, and the following quotation from page 19 of his book will do him justice on this subject:

"After mature deliberation, we have come to the conclusion, that the cause in congental as well as acquired club-foot is pre-eminently defective innervation, and there is truly no reason why derangements in the nervous system should not take place in the fœtus as well as in the new-born child. In club-foot, the tibial nerve is the bearer of the difficulty, as must be inferred from the experiments of Bonnet." * *

"All forms of varus are caused by either muscular contraction or motor paralysis, and the individual bones of the foot yield only so much in their respective positions as they are forced to do, by the abnormal muscular traction and the

---

power that its antagonists cannot extend it, the more powerful muscle soon becomes inextensible and it settles into the function of a ligament, holding firmly the points to which it is attached; the muscular tissue gradually becoming atrophied, and while the size of the muscle diminishes, its hardness increases.

This is the state of the soleus in extreme Talipes equinus. The upper end is attached to the tibia and fibula, and when the calcaneum is elevated as far as its ligaments and bony connexions will permit, the soleus can contract no farther, and if not lengthened by an opposing power it at length becomes hard and yielding. This result is prevented in the gastrocneimus by its attachment to the femur whose movements keep this muscle active and extensible. After the soleus has become rigid from *immobility*, the gastrocnemius continues to have *mobility*, and therefore it preserves its extensibility. It is not that it draws less, but that it never acquires a stationary contraction, and thefore never comes into an unyielding condition.

Disproportionate weakness of the flexors of the foot with anchylosis of the knee joint would probably result in equal extreme contraction and consequent rigidity of gastrocnemius and soleus alike.

This explanation entirely destroys the value of Dr. Pancoast's method of dividing the soleus, instead of dividing the tendo Achillis, in permanent elevations of the heel.

superincumbent weight of the body. Being held for some-time and acted upon in the preternatural position, they gradually mould themselves accordingly, and become consequently malformed."

In the opposite pathological view, it is claimed by that careful observer, Mr. Richard Barwell, that it is not usually spasm of the stronger, but paralysis of the weaker muscles, which lies at the foundation of deformity, and in support of this view he refers to the common experience that in Talipes the temperature is generally low, while in spasm it is generally high. (*Loco citate* page 19.) "Infants, as is well known, are subject to convulsions, and it is averred that sometimes one or more muscles which have during the attack drawn the limb into malposture, do not recover from the contraction, but continue to keep the limb distorted. * * The state should be one of persistent, unvarying spasm, powerful enough to overcome the antagonistic healthy muscles, and permanent enough to produce lasting change of form. Such condition does not only never come under our notice, but it is, I believe, pathologically impossible. There are no doubt a few cases of peculiar paralysis of the voluntary power over the muscles, while the excito-motory function continues; and in the spasm of the whole set, the strongest organ will of course predominate. Voluntary power is as much used to control as to excite. The paralysis of this power is as much evidenced by violent and uncontrolable spasm as by incapability of subordinate movement. In my experience, such state seldom continues long, unless there be cerebral disease or deficiency, but terminates within a limited period in death or complete recovery, or in simple paralysis in one set and perfect restoration of power in another set of muscles." * * * "Laryngismus stridulus, or false croup, is attributed by some to spasm of certain muscles, while by other authorities, and I believe with more reason, it is considered as paralysis of a different pair. Let it be observed, also, that the squint which may come and go with other symptoms of brain mischief, or may be a permanent affliction, is certainly to be more rationally regarded as want of action in the outer rectus, which appropriates the whole of one nerve, (the sixth), than as spasm of the inner rectus, whose nerve supplies four other muscles of the eye and appendages. Certain also it is, that some congenital deficiencies of the nervous system, whereof club-foot and club-hand are pretty constant accompaniments, as acephalosis, etc., etc., may, indeed must, produce paralysis; but there is no evident connection between such deformity and spasm." p. 23.

"Altogether, there can be no doubt that paralysis is very much more frequently the cause of club-foot than the opposite condition. * * * The morbid contraction of a muscle, or set of muscles, is hardly ever violent enough or persistent enough to cause a permanent alteration in the shape of the foot while the opposers remain active."

"The muscles, while healthy, are always kept at a degree of tension by tonic contraction, but when any one organ or set of organs has lost its power, the opposers draw the limb in the opposite direction by virtue of that constant and elastic sort of force. For a long time after the commencement of the paralysis there is nothing whatever wrong with the active muscles, but after they have been allowed to become thus short for a certain period they begin to adapt themselves to the shortened condition, and still further contracting as they meet with no resistance, determine at last changes of form in other structures, and so produce permanent deformity."

The clearness with which the points are here made justifies the length of the quotation.

TREATMENT.

It is believed that a careful consideration of the nature and pathology of the different varieties of Talipes, as explained in the preceding pages, will afford the foundation for clear ideas of the indications of treatment, whether preventive or curative. The plans and expedients for meeting these indications are now the earnest study of those interested in this branch of surgery. No words of mine can be more appropriate than those of Barwell (p. 25):

"It is not to be imagined that when the limb has yielded in the direction of the healthy muscles the sickly ones can recover sufficiently quick or entirely to restore, by their unassisted might, the proper balance of the foot. The weakened muscles want assistance, and the way to render this, in the manner which shall best aid them to overcome the deformity, and to recover from the paralyzed or enfeebled condition, is the problem which surgeons should endeavor to solve."

It is one of the points showing the impossibility of practically and completely separating medicine from surgery, and the different branches of surgery from each other, that in those cases of paralysis, previous to the occurrence of obvious deformity, the disease would be said to be in the department of medicine, though mechanical or chirurgical means are necessary to prevent the occurrence of deformity, and afterwards, when the deformity places the disease fairly in the department of surgery, the best period for surgical treatment has been allowed to pass by, because the case was in the department of medicine.

The physician must study surgery, and the surgeon must study medicine.

Whoever has examined a case of club-foot by taking hold of it with his hands, may have thought, that if he only had a machine that would take hold of the foot as firmly and yet as tenderly as does the hand, without relinquishing its grasp, and yet pulling yieldingly but persistently, and without tiring out, he could cure any case. The defect of every metallic apparatus is, that while it grasps the foot firmly enough, it pulls unyieldingly, without that distribution of force among all the distorted joints which is effected by the hand. They are most of them intended to act chiefly upon the tibio tarsal joint, while the most careless inspection of any species of Talipes, except one of simple Talipes equinus, will show that the distortion of this joint is a minor element in the case.

That an adequate substitute for the hand is a desideratum not yet furnished to the public, is sufficiently proved by the words of Dr. Bauer (p. 23). "There is no mechanical apparatus, however ingeniously constructed, which could be substituted for the hand in the treatment of Talipes, with any approximate degree of efficiency. In fact, if we could, without interruption, employ the hand as a mechanical agent, we should relieve most obstinate forms of Talipes, which *too frequently* withstand our mechanical appliances." This is an estimate of the importance of some substitute for the hand, with an expression of hopelessness as to its attainment.

On the other hand, Dr. Gross, in his great work on Surgery, vol. II, p. 1011, is well enough satisfied with our present attainments in the art, neither desiring nor expecting any improvements. He says, "It is, perhaps, not going too far to affirm that these topics (club-foot, etc.,) are as well understood now as they ever will be."

Dr. Bauer again places this estimate upon our present attainments (p. 28). "They (mechanical appliances) possess no curative virtues, but retain the foot in the position in which tenotomy and the acting hand left it."

It is believed that in the course of these pages a process will be explained which is a pretty adequate substitute for the hand.

The earlier experimenters in this art seem to have relied chiefly upon wood and iron as substitutes for the hand, but so generally did they occasion ulcerations of the prominent parts that the art made no important progress until the introduction of subcutaneous section of the tendons by Stromeyer, in 1831. In a large proportion of the cases of Talipes, including all the species *equinus*, the division of the tendon Achilles permits an immediate improvement in the position of the foot, and facilitates the further reduction of the distortion of the joints of the tarsus. This tendon had been cut at various times before Stromeyer, by making an open wound, but this procedure could never be generally adopted. Dr. H. G. Davis, in his report on Deformities, in the Transactions of the National Medical Association for 1863, quotes Isaac Mincius as having divided it in 1685; Thellenius, in 1784; Sartorius, in 1806; Michaelis, in 1809; Delpech, in 1816; but none of these men could think of so simple an expedient as passing in a small knife at a point distant from the tendon, and so dividing it that the incision through the skin should heal without suppuration. It is commonly recommended, to puncture the skin, with a sharp pointed bistoury, upon the inner or tibial side of the tendon, opposite the internal malleolus, or higher, if the heel is very much elevated, and having withdrawn this to pass a probe pointed bistoury between the tendon and the tibia, and while the tendon is made very tense by the hand of an assistant holding the foot, to cut the tendon by pressing the fingers upon it, thus crowding it upon the knife. If any shreds remain undivided the fact is known by the failure of the heel to come down, and the bistoury is again partially withdrawn and passed under them, when they are divided by the same process by which the main portion of the tendon was cut. The reason for passing the knife on the tibial side of the tendon is the less danger of wounding, by the point of the knife, the posterior tibial artery, which lies upon the inner side, and the same reason exists for cutting toward the skin instead of passing the knife between the tendon and the skin and cutting toward the bone. A small piece of plaster laid over the minute incision is all the dressiug that is necessary.

It is common to describe instruments peculiarly constructed for this purpose, but they are unnecessary. Many of the instruments made for tenotomy are too delicate.

Apparatus for extension is immediately applied by some, but in order to secure a union of the divided ends of the tendons by organizing exudations it may be more safe to postpone this for a few days, and then to make the extension very gradually. It is not known that the tendo Achilles, divided subcutaneously in early life in the human subject, has ever failed to unite, but in an experiment which I made some years ago, upon a dog, the divided tendo Achilles united only by shreds of its investing sheath, which indeed may never have been divided.

It is suspected that the uniform success of division of the tendo Achilles, as introduced by Stromeyer, gave an unmerited estimate of the importance and utility of the division of tendons and muscles in general. A reaction in this estimate has led many to discontinue the practice of dividing tendons, except in rare cases of remarkable obstinacy, while others seem still to believe in tenotomy with undiminished zeal.

Among the former is Mr. Richard Barwell, of London, who says in the preface to his little book, "I studied these maladies from the orthopedic point of view, and while tenotomy was almost a novelty in England, was so charmed with the easy change of form, which after such an operation could be produced in most distortions, that I became an almost enthusiastic admirer of the procedure. After, however, following up carefully a large number of these cases, I was pained to find in how many of them the deformity more or less returned, in how many a different, an opposite distortion supervened; while power over the limb was actually injured or destroyed in so large a majority of instances that its retention appeared absolutely exceptional."

This language sounds very much like that of one temporarily thrown out of balance by an extreme reaction in opinion, instead of stopping at the safe middle point.

The latest published opinions on the other side, are those of Dr. Bauer, (p. 34 of the little book already referred to,) who says, "The active forms of valgus necessitate the division of the contracted peroneus muscles, or of the whole group of the abductors as the case may be. This is at least indispensable in inflammation of the tibio tarsal articulation. * * * It is difficult to steady the articulation with mechanical appliances in paralysis of the entire motor apparatus of the foot, but it is completely impossible to do so when the malposition of the latter is maintained by retraction of the peronei muscles. We at least, have never succeeded by any of the devised mechanical auxiliaries. Meanwhile the deformity increases and gradually compromises the bones of the tarsus. Between the two evils we have to choose, and we think that division of the contracted tendons is the lesser."

Now it is the division of these tendons which, like the peronei, run in long ligamentous grooves along the tarsus, which is most objected to. It is claimed that the function of these muscles is often permanently suspended by division, either by not uniting, or by adhering to their sheaths, so as no longer to be able to act upon the bones into which they are normally inserted.

Mr. Wm. Adams, of London, has been investigating this subject during the last few years, and has dissected twelve feet in which tenotomy had been performed. The results of these investigations have been published under the title, "On the Reparative Process in Human Tendons." Mr. Barwell has reduced these results to tabular form, which is here quoted.

*Table from "Boswell on Club-Foot," Edit.* 1863—*Analyzed from "Adams on the Reparative Process in Human Tendons."*

| No. Cases. | Tendons Divided. | Results Observed. | Time lived after operation. |
|---|---|---|---|
| I. | Tendon Achilles,<br>Tibialis Anticus, | Non-union of Tibialis Anticus, | 4 days. |
| II. | Tendon Achilles,<br>Tibialis Anticus,<br>Tibialis Posticus<br>Flexor long. dig. | Non-union of Tibialis Anticus.<br>Non-union of Flexor long. digit. | 11 days. |
| III. | left. Tendon Achilles,<br>Tibialis Posticus | Tibialis Posticus adherent to the bone.................. ...... | 23 days. |
| | right. Tendon Achilles,<br>Tibialis Posticus<br>Tibialis Anticus, | Tibialis Posticus was supposed to be but was not, divided. | 30 days. |
| IV. | Tibialis Posticus,<br>Flexor long. dig. | Union to all surrounding parts.<br>Non-union, held together by shreds of sheath to which other parts also adhered........... .... | 18 days. |
| V. | Tendon Achilles.<br>Tibialis Anticus,<br>Tibialis Posticus<br>Flexor long. dig. | Tibialis Posticus and Flexor longus digitorum adhered together and to the bone .......... | 6 weeks. |
| VI. | Tendon Achilles,<br>Tibialis Anticus.<br>Tibialis Posticus<br>Flexor long. dig. | Tibialis Anticus and Flexor long, dig. adhered together and to the bone; ends of tibialis ant. hung together by shreds of sheath. | 6 weeks. |
| In the 5 next cases in Mr. Adams' work the Tendon Achilles only was divided. | | | |
| XII. | Tendon Achilles,<br>Tibialis Posticus<br>Flexor long. dig. | Non-union of Tibialis Anticus.<br>Neither retraction nor extension of the Flexor longus digit. | Several yrs |

*Analysis of the Preceding Table.*

Division of the Tendo Achilles, 12 cases; united in 12.

Division of the Tibialis Anticus, 4 cases; united in 1; not united in 3; adhered to surrounding parts, equally destroying the function of the muscle, in 1.

Division of the Tibialis Posticus, 7 cases; not divided in 1; united in 3; not united in 3; adherent to bone or surrounding parts, suspending the function of the muscle, in 3; that is, in all the cases of non-union.

Division of the Flexor Longus Digitorum, 5 cases; united in 1; not united in 4; adherent to surrounding parts, (among the cases classed non-union,) 2.

From this analysis we may well hesitate before dividing any tendon about the foot, except the Tendo Achilles. If the result in these cases is of any value, the division of these tendons should only be practiced in instances in which, from per manent loss or paralysis of the opposing muscles, a permanent loss of muscular contraction is desirable in the muscles whose tendons are to be divided.

The following interesting observations and experiments by Dr. L. T. Hewins, of Loda, Iroquois co., Ill., show the influence of young ag› upon the activity of cicatrix formation to connect the divided ends of tendons, or to pull them together.

Upon a dog four years old, he failed. Upon dogs ten days, and three months old, he succeeded, after removing portions of tendons. He also succeeded perfectly upon a rabbit. He observed the reproduction of tendon, or substitute for it, in the extensor digitorum manus, in one man 35 years old, three-fourths of an inch having sloughed off, and in another man, aged 38, half an inch having been lost by sloughing.

These latter cases were successes under difficulties, the wounds being open and granulating, and presenting the conditions and favoring agglutination of the tendons to the bones and other surrounding parts. The influence of motion in elongating adhesions and reducing shapeless masses of newly organized material to the shape and function of tendon, whether permanent or temporary, by its gradual shortening and disappearance, is well illustrated.

LODA, ILLS., Sept. 12, 1862.

Divided the tendon of a healthy dog, about four years old, corresponding to the Tendo Achillis in man. Removed a section of the tendon so as to be sure if I could get reproduction of tendon in an animal of that age. Dressed the limb with splints and rollers to prevent motion.

Sept. 20th.—Removed dressing from the limb. External wound healing kindly; no evidence of growth of tendon.

Oct. 2d.—Examined limb; no evidence of reproduction; fascia both superficial and deep-seated, are quite adherent to the divided ends of the tendon.

Oct. 15th.—Removed dressing from limb; no elongation of tendon; fascia and tendon uniting; fascia more firm than at former examination, and evidently thickening.

Dec. 1st.—Examined the divided tendon; find no evidence of growth in length of tendon; fascia have united with divided ends of the tendon, to form a common link between those parts. The dog walks with a hobbling gait.

Sept. 12th, 1863.—One year after the division of the tendon in the above case, there is no evidence of reproduction of tendon; the divided ends may be felt through the integument and the fascia is very firm. Dog has a hobbling gait and is permanently lame.

Sept. 13th.—Divided the tendon in a dog about ten days old, corresponding to the one divided in the former case, and a portion of the tendon removed. Dressed the limb to keep at rest; dog seemed entirely healthy.

Sept. 20th.—Dressed the limb. There is evident prolongation of tendon.

Oct. 2d.—Dressed the leg. Tendon manifestly extending, so as nearly to unite.

Oct. 12th.—Tendon not yet united, kept on dressing.

Oct. 23d.—Tendon not completely united, but the divided ends approaching each other.

Nov. 15th.—Examined the leg; found the tendon entirely united, having a good degree of firmess; dog walks without halting.

Dec. 25th.—Divided tendon seems as strong as undivided one of the other leg; dog walks without limping.

Feb. 2d, 1864.—Divided tendon of a dog three months old. Dressed, after removing a portion of the tendon, so as to keep from motion.

Feb. 10th.—Dressed the leg; wound in integument healing kindly; evident formation of new tendon.

Feb. 20th.—Dressed the leg; tendon still growing in length.

March 2d.—Dressed the leg; found divided ends of tendon approaching each other.

April 1st.—Tendon fully formed and pretty firmly united; wound has healed kindly. Dog walks well.

March 3d.—Divided the tendon in the leg of a rabbit, and dressed to keep motionless.

March 10th.—Dressed the wound; looks well, tendonous organization evidently going on well.

March 20th.—Tendon elongated; union hopeful.

March 30th.—Tendon fully formed, but soft.

April 15th.—Tendon fully formed and more firm. Animal walks well. This animal seemed very healthy.

Nov. 4th, 1862.—D. S., (a German by birth,) a healthy man, aged 35 years, had the extensor tendon of the middle finger on the left hand divided by a corn-knife. Wound was neglected about 14 days, by which time the tendon had ulcerated and about three-fourths of an inch had sloughed out, when he applied to me for treatment. Dressed the hand and kept the finger extended and at rest; attempted to subdue inflammation in the hand, which was at the time extensive, as soon as possible, and to arrest ulceration of tendon and its necessary distinction. After twelve weeks new tendon had been produced to supply the waste made by previous ulceration, and the finger restored to its normal action.

April 15th, 1864.—Mr. J. D., a man aged 38 years, had his index finger on left hand seriously injured by contused wound from a hand-car. Ten days afterward he applied to me for treatment. I found about half an inch of the extensor tendon of the finger sloughed off; have dressed and watched the finger carefully to this date, June 2d, and by this time a new tendon has formed, but is soft. I think we shall have a good finger. L. T. H.

(*To be continued.*)

## Figures and Tables

**Figure 4. f1:**